# Supplementary material for: Students and examiners perception on virtual medical graduation exam during the COVID-19 quarantine period: A cross-sectional study
Source: PLoS One. 2022 Aug 19;17(8):e0272927. doi: 10.1371/journal.pone.0272927 (PMC9390930; doi:10.1371/journal.pone.0272927)
Supplement: S1 File — (PDF) [file pone.0272927.s001.pdf]

Final assessment examiner feedback

Your opinion and experience with us are very important because your feedback will improve the quality of assessment for future years. It will take less than 5 minutes to complete the survey. All information you provide will remain anonymous and only aggregate information will be shared publicly and with higher education officials.

* Required

1. Do you wish to participate *

Mark only one oval.

- Yes
- No

2. Gender *

Mark only one oval.

- Male
- Female

3. You were responsible for questions from -------- department *

Mark only one oval.

- Medicine
- Surgery
- Obstetric &Gynecology
- Pediatrics

4. 1. You were clearly informed about the assessment process *

Mark only one oval.

	1	2	3	4	5	
Strongly disagree	◯	◯	◯	◯	◯	Strongly agree

5. 2. During the assessment, the technical support by cohost was adequate *

Mark only one oval.

	1	2	3	4	5	
Strongly disagree	◯	◯	◯	◯	◯	Strongly agree

6. 3. During the assessment, health regulation, physical distancing, and mask wearing on the day of the exam (at college) was adequate. *

Mark only one oval.

	1	2	3	4	5	
Strongly disagree	◯	◯	◯	◯	◯	Strongly agree

7. 4. Exam duration was acceptable. *

Mark only one oval.

	1	2	3	4	5	
Strongly disagree	◯	◯	◯	◯	◯	Strongly agree

8. 5. Assessment reflected real clinical practice. *

Mark only one oval.

	1	2	3	4	5	
Strongly disagree	⬭	⬭	⬭	⬭	⬭	Strongly agree

9. 6. The question reflected proper sampling from the curriculum *

Mark only one oval.

	1	2	3	4	5	
Strongly disagree	⬭	⬭	⬭	⬭	⬭	Strongly agree

10. 7. You were satisfied with the provided assessment checklist *

Mark only one oval.

	1	2	3	4	5	
Strongly disagree	⬭	⬭	⬭	⬭	⬭	Strongly agree

11. 8. The online assessment could assess clinical competence. *

Mark only one oval.

	1	2	3	4	5	
Strongly disagree	⬭	⬭	⬭	⬭	⬭	Strongly agree

12. 9. Organization of the whole process met your expectation *

Mark only one oval.

	1	2	3	4	5	
Strongly disagree	⬭	⬭	⬭	⬭	⬭	Strongly agree

13. What did you like best in the assessment? *

____
____
____
____
____

14. What did you dislike? (mention one item of dislike). *

____
____
____
____
____

15. Suggestions for improvement. *

Thank you for taking the time and provide your feedback
