# Supplementary material for: Students and examiners perception on virtual medical graduation exam during the COVID-19 quarantine period: A cross-sectional study
Source: PLoS One. 2022 Aug 19;17(8):e0272927. doi: 10.1371/journal.pone.0272927 (PMC9390930; doi:10.1371/journal.pone.0272927)
Supplement: S2 File — (PDF) [file pone.0272927.s002.pdf]

Type your text

Final assessment student feedback

Your opinion and experience with us are very important because your feedback will improve the quality of assessment for future years. It will take less than 5 minutes to complete the survey. All information you provide will remain anonymous and only aggregate information will be shared publicly and with higher education officials.

* Required

1. Do you wish to participate? *

Mark only one oval.

- ( ) Yes
- ( ) No

2. Gender *

Mark only one oval.

- ( ) Male
- ( ) Female

3. 1. You have received comprehensive instructions on the way of online assessment *

Mark only one oval.

	1	2	3	4	5	
Strongly disagree	( )	( )	( )	( )	( )	Strongly agree

4. 2. There was sufficient communication between school administration and student *

Mark only one oval.

	1	2	3	4	5	
Strongly disagree	◯	◯	◯	◯	◯	Strongly agree

5. 3. The format of the online assessment was acceptable. *

Mark only one oval.

	1	2	3	4	5	
Strongly disagree	◯	◯	◯	◯	◯	Strongly agree

6. 4. The examiners were professional *

Mark only one oval.

	1	2	3	4	5	
Strongly disagree	◯	◯	◯	◯	◯	Strongly agree

7. 5. The assessment questions were clear *

Mark only one oval.

	1	2	3	4	5	
Strongly disagree	◯	◯	◯	◯	◯	Strongly agree

8. 6. The conducted assessment can assess clinical skills *

Mark only one oval.

	1	2	3	4	5	
Strongly disagree	⬭	⬭	⬭	⬭	⬭	Strongly agree

9. 7. The time allocated for each question (medicine, surgery, obe&gyn, pediatrics) was enough. *

Mark only one oval.

	1	2	3	4	5	
Strongly disagree	⬭	⬭	⬭	⬭	⬭	Strongly agree

10. 8. Did you face connection problems during the assessment? *

Mark only one oval.

⬭ Yes *Skip to question 11*

⬭ No *Skip to question 13*

Untitled Section

11. The problem was about *

Mark only one oval.

- ( ) Electricity shutdown *Skip to question 11*
- ( ) Internet problem *Skip to question 11*
- ( ) Both *Skip to question 11*

12. Was the problem solved *

Mark only one oval.

- ( ) Yes *Skip to question 13*
- ( ) No *Skip to question 13*

13. 9. Overall, You were satisfied with the assessment process. *

Mark only one oval.

	1	2	3	4	5	
Strongly disagree	( )	( )	( )	( )	( )	Strongly agree

14. What did you like best in the assessment? *

____

____

____

____

____

15. What did you dislike?. *

16. Suggestions for improvement. *

Thank you for taking the time and provide your feedback
