# Supplementary material for: Students and examiners perception on virtual medical graduation exam during the COVID-19 quarantine period: A cross-sectional study
Source: PLoS One. 2022 Aug 19;17(8):e0272927. doi: 10.1371/journal.pone.0272927 (PMC9390930; doi:10.1371/journal.pone.0272927)
Supplement: Attachment — Submitted filename: Response to reviewers.docx [file pone.0272927.s003.docx]

Vijayalakshmi Kakulapati,
Academic Editor
PLOS ONE

11^th^ May, 2021

Dear Vijayalakshmi Kakulapati**,**

On behalf of the co-authors I would like to thank you for inviting us to resubmit a revised version of our manuscript entitled “**Students and examiners perception on virtual medical graduation exam during the COVID-19 quarantine period: a cross-sectional study**”. Please see our point-by-point response to the comments raised by the editor and reviewers below. We have modified the manuscript according to suggestions made by the editor and reviewers and believe that the manuscript has significantly improved. We hope it will now be suitable for publication in the PLOS ONE Journal.

Yours sincerely,

Nazdar Alkhateeb

Assistant Professor, PhD Medical Education

Hawler Medical University

**Responses to editor and reviewers’ comments**

|  | Comments | Response |
| --- | --- | --- |
| **Editor** | 1. Please ensure that your manuscript meets PLOS ONE's style requirements, including those for file naming. | Thank you for your comment. The manuscript prepared according to the journal style (figures and tables) |
|  | 2. Please provide additional details regarding participant consent. In the Methods section, please ensure that you have specified (1) whether consent was informed and (2) what type you obtained (for instance, written or verbal). If your study included minors, state whether you obtained consent from parents or guardians. If the need for consent was waived by the ethics committee, please include this information. | An additional subtitle “Ethical consideration” added to methods section and include more details on the informed written consent.  All the participants of the study were adults |
|  | 3. Your ethics statement should only appear in the Methods section of your manuscript. If your ethics statement is written in any section besides the Methods, please move it to the Methods section and delete it from any other section. Please ensure that your ethics statement is included in your manuscript, as the ethics statement entered into the online submission form will not be published alongside your manuscript. | The ethics statement moved to Methods section. |
|  | 4. Please review your reference list to ensure that it is complete and correct. If you have cited papers that have been retracted, please include the rationale for doing so in the manuscript text, or remove these references and replace them with relevant current references. Any changes to the reference list should be mentioned in the rebuttal letter that accompanies your revised manuscript. If you need to cite a retracted article, indicate the article’s retracted status in the References list and also include a citation and full reference for the retraction notice. | Many thanks for informing me. I found a retracted paper had been cited mistakenly.  Reference no. 3 changed to another reference entitled  ” Huber SG, Helm C**. COVID-19 and schooling: evaluation, assessment and accountability in times of crises—reacting quickly to explore key issues for policy, practice and research with the school barometer**. Educ Assessment Eval Account. 2020;32: 237–270. doi:10.1007/S11092-020-09322-Y/FIGURES/7. PMID: 32837626; PubMed Central PMCID: [PMC7286213](http://www.ncbi.nlm.nih.gov/pmc/articles/pmc7286213/)” |
| **Reviewer #1** | 1. This article presents a description of the online assessment adopted at Hawler Medical University/Iraq during the lockdown period of the COVID-19 pandemic, mainly for final year medical students, followed by an evaluation based on questionnaires for both examiners and students. Afterwards, a discussion of the outcomes of the questionnaire is presented. | No comment |
|  | 2. One cannot classify this paper as a rigorous study. The outcomes, results and conclusions are quite trivial to the extent that one could have easily predicted them. Not to add that authors themselves found that they had almost the same results with similar studies (lines 261-263 and 295-297). | Thank you for your comment. The study was done at the beginning of the COVID-19. All the world specially the developing countries with low resources and limited technology information were struggling to find a way for assessing final year medical students, it was necessary to share the experience of conducting an online assessment in such circumstances. |
|  | 3. The description of the online assessment should be complemented by clear and descriptive. This not the case here. Lines 116-120 that are supposed to explain the distribution of students are not well presented, nor the figure 1. I wasn’t able to map the information presented in the paragraph to the Figure. Not to add that the figure is ‘weak’. Moreover, Figure 2 is simply not necessary; I cannot see any added value; I would remove it. Regarding Figure 3, it is a bit vague, and no explanation in the caption. “Examination” is written as “Eamination”. | Thank you for your comment. We agree that the figure was not very clear, we revised the paragraph (line 116-120) to make it clearer. And added a figure legend to both figure 1 and 3. Figure 2 was removed.  The revised part is with track changes |
|  | 4. As for the statistical part, the number of the participants as mentioned in line 168, (75 students and 54 examiners) is considered not a sufficiently large one to conduct a thorough study. And more importantly, the interpretation of p-value; Are the p-values presented in Table 1 considered ‘good’ enough from a statistical perspective? | As the reviewer mentioned the number of the participants were 75 students and 54 examiners  The sample size calculation mentioned in line 71-80, the response rate among students and examiners were 69.4% and 88.5% respectively which is considered high.  The p value in Table 1 showed non-significant differences. |
|  | 5. The questionnaire is not comprehensive and questions are very general. I would expect some very specific questions related to the quality/clearness of the X-rays and other pictures/figures, diverse type of tests (blood, urine…), comprehensiveness of case studies… does the scope of the questions cover a substantial area of knowledge in each discipline? | The reviewer was absolutely right regarding the questionnaire questions being very general and missed specific questions.  This was the case because the aim of this study was to evaluate the first online final year assessment which was conducted in a low resource country, and to help administration for future steps and at the end to share the experience of the implemented plan with the medical education community. |
|  | 6. Conclusions and recommendations” section is very small; I would merge them with “Discussions | The conclusion section was merged with the discussion |
|  | 7. Line 22: “while” instead of “with”. Lines 23: “…aims at sharing…” instead of “…aims to share…”. Same applies to lines 57 Line 31: usage of “had been” is to be revised. Line 51: “have been” to be removed. Line 55: “Our medical school”, please specify since it is the first instance, so that reader does not have to refer to the affiliations. Line 125: “were” instead of “was” in both instances. Line 131, a comma to be inserted after “faculties”. Line 132, “proficient” instead of “efficient” Usage of “had been” to be reviewed in lines 161-166. In line 206, “Around half (48.1%) were males, and the male: female ratio was 0.92: 1.” Redundancy! either part of the sentence is to be kept not both. Line 235, “presented” instead of “present” Line 260, “have been” to be removed. Line 264, “has” instead of “have” Line 265, a semicolon instead of a comma. In general, English should be thoroughly reviewed | Many thanks for your valuable corrections. All the suggested changes were done. |
| **Reviewer#2** | 1. In this work, the authors conducted a research work that included online questionnaire to assess virtual medical graduation exam effectiveness from both examiners’ and students’ perspectives. | No comment |
|  | 2. Add more details on reasons why third of students were not satisfied with the online exam. | Thank you for your helpful comment. Reasons are added as following paragraph  “On the other hand, concerns about the number of questions used, the marks allocated for the final assessment, technical problems and poor nonverbal communication on videoconferencing were mainly disliked by a certain fraction of students and might have contributed in their non-satisfaction. According to Mak and his colleagues it is hard to gain control on the full range of nonverbal communication through the online platform due to screen size as some students used their mobile phones, position of the student on the screen as well as impaired video quality due to internet problems[23]. This was also found in our students as a good percentage of them used mobile screens for joining the exam. ” |
|  | 3. Use of additional methods including interviews can be helpful to collect new /augment existing data. 4. Researchers seemed to rely more on quantitative methods. I recommend to include qualitative methods as it can capture different types of data/interactions that are hard to collect with questionnaires. | Thank you for raising this point. we relied mainly on quantitative data but open ended questions was also used and thematic analysis was performed to extract data (Table3-6). |
|  | 5. Researchers need to add more discussion on other tools/technologies that can be used other than Zoom for examination. | The requested discussion was added as the following paragraph  **“**Technological development and accessibility to variety of digital tools have supported the education process in COVID-19 era throughout the globe. Zoom, Google meet, Microsoft team, WebEx,…etc are examples. Overall, these tools enabled sharing clinical photographs, laboratory results and imaging using the screen-sharing feature, and facilitated the assessment of clinical reasoning and data interpretation domains via examiner questioning synchronously. Problem-based questions enabled the assessment of the clinical reasoning and higher-order thinking skills [16,17]. Hawler Medical University used Zoom as a tool for the final year assessment as the staff and students had experienced it during the early COVID-19 period.” |
