# Supplementary material for: Students and examiners perception on virtual medical graduation exam during the COVID-19 quarantine period: A cross-sectional study
Source: PLoS One. 2022 Aug 19;17(8):e0272927. doi: 10.1371/journal.pone.0272927 (PMC9390930; doi:10.1371/journal.pone.0272927)
Supplement: Attachment — Submitted filename: Response to reviewer second round.docx [file pone.0272927.s004.docx]

Vijayalakshmi Kakulapati,
Academic Editor
PLOS ONE

13^th^ July, 2022

Dear Vijayalakshmi Kakulapati**,**

On behalf of the co-authors I would like to thank you for inviting us to resubmit a second revised version of our manuscript entitled “**Students and examiners perception on virtual medical graduation exam during the COVID-19 quarantine period: a cross-sectional study**”. Please see our point-by-point response to the comments raised by the editor and reviewers below. We have modified the manuscript according to suggestions made by the editor and reviewers and believe that the manuscript has significantly improved. We hope it will now be suitable for publication in the PLOS ONE Journal.

Yours sincerely,

Nazdar Alkhateeb

Assistant Professor, PhD Medical Education

Hawler Medical University

**Responses to editor and reviewers’ comments**

|  | **Comments** | **Responses** |
| --- | --- | --- |
| **Editor** | 1. Need to check language corrections | Thank you for your comment. The manuscript underwent language editing and grammar mistakes corrected |
|  | 2. Please review your reference list to ensure that it is complete and correct. If you have cited papers that have been retracted, please include the rationale for doing so in the manuscript text, or remove these references and replace them with relevant current references. Any changes to the reference list should be mentioned in the rebuttal letter that accompanies your revised manuscript. If you need to cite a retracted article, indicate the article’s retracted status in the References list and also include a citation and full reference for the retraction notice. | Many thanks for informing me.  Based on cross reference meta data link references function, some references have been removed and some were added  The removed references were:  Ref. no. 1  Pokhrel S, Chhetri R. A Literature Review on Impact of COVID-19 Pandemic on Teaching and Learning. High Educ Futur. 2021;8: 133–141.  Ref. no.4  Murphy J. Assessment in medical education. Ir Med J. 2007;100: 356. Available: http://archive.imj.ie//ViewArticleDetails.aspx?ContentID=3620  Ref.no.11  Sabzwari S. Rethinking Assessment in Medical Education in the time of COVID-19. MedEdPublish. 2020;9: 1–6.  Ref. no. 12  Shehata MH, Kumar AP, Arekat MR, Alsenbesy M, Mohammed Al Ansari A, Atwa H, et al. A toolbox for conducting an online OSCE. Clin Teach. 2021;18: 236–242.  Ref.no.14  Al-Mendalawi MD. Teaching Paediatrics in Iraq Amid the COVID-19 Pandemic. Sultan Qaboos Univ Med J. 2020;20: e408  Ref. no.19  Rezaei H, Haghdoost A, Javar HA, Dehnavieh R, Aramesh S, Dehgani N, et al. The effect of coronavirus (COVID-19) pandemic on medical sciences education in Iran. J Educ Health Promot. 2021;10. doi:10.4103/JEHP.JEHP_817_20  Ref. no.24  Pettit M, Shukla S, Zhang J, Sunil Kumar KH, Khanduja V. Virtual exams: has COVID-19 provided the impetus to change assessment methods in medicine? Bone Jt open. 2021;2: 111–118.  Ref. no.26  Derbel F. Technologically-Capable Teachers in a Low-Technology Context.  The following references were added  Choi B, Jegatheeswaran L, Minocha A, Alhilani M, Nakhoul M, Mutengesa E. The impact of the COVID-19 pandemic on final year medical students in the United Kingdom: a national survey. BMC Med Educ. 2020;20: 206.  Guangul FM, Suhail AH, Khalit MI, Khidhir BA. Challenges of remote assessment in higher education in the context of COVID-19: a case study of Middle East College. Educ Assessment, Eval Account. 2020;32: 519–535.  Yapa HM, Bärnighausen T. Implementation science in resource-poor countries and communities. Implement Sci. 2018;13: 1–13. |
| **Reviewer#2** | 1. The authors addressed my comments and provided clear feedback. | No comment |
|  | 2. The qualitative analysis might need more refinement/details. The authors mentioned conducting thematic analysis but they didn't reveal patterns/themes identified in the open ended questions. How it helped and what more information it provided? | Thank you for your helpful comment.  Table 3 and table 6 which present thematic analysis of the open-ended questions were modified, themes and subthemes were added to it.  A paragraph was added in the result section as follows  “Table 3 shows the main themes and subthemes that emerged from the students’ perception on Pros and Cons of the online assessment. The two main areas highlighted in the Pros were the question and format of the exam, and the professional behavior of the examiners. The students mostly liked the type of the questions, being practical and covering the common clinical and emergency cases reasonably (34.6%). On the other hand, the two main themes that emerged in the Cons were related to administration issues, and question and format of the assessment. The major areas that students disliked were as follows: long waiting time and delay (22.7%), unfair distribution of marks (22.7%), connection problems (20%). Other subthemes are presented in detail in Table 3.” |
